# Disease modifying therapies for multiple sclerosis: benefit and acceptability

**DOI:** 10.12968/bjnn.2022.18.Sup3.S16

**Published:** 2022-07-01

**Authors:** Joanna Harrison, James Hill, Karen Palmer

**Affiliations:** 1 Health Technology Assessment Unit, University of Central Lancashire; 2 Research and Development, Lancashire and South Cumbria NHS Foundation Trust

## Abstract

Disease modifying therapies are available for the treatment of relapse remitting multiple sclerosis, making relapses less common and severe. A Cochrane systematic review was undertaken to compare their benefit and acceptability. This article summarises and appraises the review evidence.

## Introduction

Multiple Sclerosis (MS) is a chronic, auto-immune disease caused by inflammatory damage to myelin in the central nervous system (Coles, 2009). MS symptoms typically manifest as the involvement of motor, sensory, visual and autonomic systems depending on the area in the central nervous system that is affected (Compston and Coles, 2008). In collaboration with Public Health England, the MS Society (2020) estimate that there are over 130 000 people with MS in the UK, and that each year nearly 7000 people are newly diagnosed, with the prevalence more than double in females compared to males.

Approximately 85% of people with MS are initially diagnosed with relapsing remitting MS (RRMS) (National MS Society, 2022), defined by periods of new or worsening neurological symptoms, lasting longer than 24 hours, and in the absence of any other cause (National Institute for Health and Care Excellence, 2016). A relapse is characterised by symptoms that commence slowly, stabilise over days or weeks and resolve in a gradual way, either completely or in-part (Coles, 2009). Management of RRMS includes treatment with disease modifying therapies (DMTs) to reduce disease activity and progression (Gallo et al, 2015). There are a wide range of DMTs available for use as either a pill, injection or intravenous infusion, each with a different mechanism of action. In 2015, a Cochrane systematic review was undertaken to compare the benefit and acceptability of DMTs (immunomodulators and immunosuppressants) for the treatment of people with RRMS (Tramacere et al, 2015).

This commentary aims to critically appraise the methods used within the Cochrane systematic review and to discuss the findings in the context of more recent clinical evidence and the implications for clinical practice.

## Methods

A multiple database search was undertaken from the date of inception to September 2014. Additional screening of included studies and relevant systematic review citations was undertaken. Only randomised controlled trials (RCTs) that compared one or more immunomodulators or immunosuppressants (all types) to different active therapies or a placebo were included. RCTs that completed the follow-up in less than six months were excluded, as were those trials that evaluated combination treatments, comparison of same drug regimens, non-pharmacological treatments and over the counter drugs.

Participants from the included trials were aged 18 years or over, with a diagnosis of RRMS according to diagnostic criteria. There were no exclusions of the participants based on sex, degree of disability or disease duration.

Screening, data extraction and assessment of bias (using the Cochrane collaboration criteria), were undertaken independently by two reviewers, with arbitration by a third reviewer. The primary outcomes for the review were the proportion of participants experiencing relapse (new or worsening symptoms), disability worsening (irreversible worsening at 3 months) and acceptability (number of participants who withdrew because of an adverse event). Secondary outcomes were the total number of serious adverse events. Pairwise conventional meta - analyses were undertaken for all primary outcomes using a random effects model. A network meta-analysis was also undertaken for relapses, disability worsening and acceptability outcomes, and a surface under the cumulative ranking curve (SUCRA) score (0-100%) was calculated to enable the ranking of interventions. To evaluate the presence of statistical inconsistency, a loop specific method was used for local networks and ‘design by treatment’ for the entire network. A classification of confidence in the estimated effect was given using grading of recommendations assessment, development and evaluation (GRADE) for network meta-analysis. Risk of bias and inconsistencies were taken into consideration when grading the overall confidence in each outcome.

## Main Review Findings

A total of 39 studies, comprising 25 113 randomised participants diagnosed with MS were included in the review. Of these studies, 24 (60%) were controlled by a placebo group, whereas 15 (40%) were a head-to-head comparison. Most of the trials assessed short-term outcomes (median follow up of 24 months). The following DMTs were evaluated: Interferon beta-1b (Betaseron), Interferon beta-1a (Avonex and Rebif), Glatiramer acetate, Natalizumab, Mitoxantrone, Fingolimod, Teriflunomide, Dimethyl fumarate, Alemtuzumab, Pegylated interferon beta-1a, Daclizumab, Laquinimod, Azathioprine and Immunoglobulins.

The risk of bias for the included studies was mixed with 51% of studies judged as high, 41% as moderate and 8% as low risk of bias. The main areas of high risk were non-blinding of participants and investigators (15 studies, 38%), incomplete outcome data (14 studies, 36%) and other biases (33 studies, 85%) such as the role and influence of the study sponsor in authorship. Over a 24-month period, local statistical inconsistency was observed in the network meta-analysis of relapse and disability worsening. There was no indication of global inconsistency within any network. Owing to the limited number of studies per comparison and statistical inconsistency, the quality of evidence was downgraded in most comparisons.

Over the 12- and 24-month periods, the network meta-analysis showed that the most effective therapies for reducing the risk of relapse in RRMS, when compared to a placebo, were Alemtuzumab, Mitoxantrone, Natalizumab and Fingolimod (Table 1 and 2). All four therapies demonstrated a clinical and statistical reduction in the risk of experiencing a relapse. Over the 12- and 24-month period, excluding Mitoxantrone and Natalizumab, Alemtuzumab showed a significantly lower proportion of participants who experienced new relapses.

Over a 24-month period, when compared to a placebo, the most effective therapies for reducing the risk of disability worsening were Mitoxantrone, Alemtuzumab, Natalizumab and Azathioprine (Table 3). All four therapies demonstrated a clinical and statistical reduction in the risk of disability worsening. Over the 24-month period, Alemtuzumab also demonstrated a significant reduction in the risk of disability worsening compared to all other therapies excluding Mitoxantrone, which showed a significant reduction in the proportion of participants who experienced disability worsening compared to a placebo and five other therapies.

The network meta-analysis showed that over a 12-month period several therapies had a significantly increased risk of treatment withdrawal because of adverse events, when compared to a placebo group. These were Teriflunomide, Peg-interferon beta-1a, Interferon beta-1a (Avonex), Interferon beta-1a (Rebif) and Fingolimod (Table 4). Over 24 months, only fingolimod had a significantly higher risk of treatment withdrawal when compared to the placebo group (risk ratio 1.69, 95% confidence interval 1.32-2.17). There was limited and unclear evidence for the difference in risk of serious adverse events when comparing all active therapies to a placebo. There was no statistically significant difference for any of the subgroup or sensitivity analyses undertaken.

## Quality of Systematic Review

The Amstar2 tool was used to assess the quality of the Cochrane systematic review, scoring it 16 out of 16 (Shea et al, 2017). Therefore, this systematic review provides an accurate and comprehensive summary of the available studies that address the question of interest.

However, other factors should be taken into consideration when interpreting the findings from this review. For example, there were limited numbers of trials that directly compared DMTs. It should also be considered that all the therapies were evaluated within the short-term (24-month follow-up period). Therefore, identifying benefit beyond this timeframe is uncertain and thus, limits the implications for practice for what is a life-long disease. Furthermore, the short-term nature of these trials does not provide enough data on serious adverse events and so makes it difficult to assess the risk related to these therapies.

## Implications for practice

The most effective DMTs for preventing clinical relapses in RRMS based on the quality of evidence required (moderate to high quality) and limited to the first 24 months of treatment are Alemtuzumab, Natalizumab and Fingolimod. Given the quality of evidence identified for the proportion of participants experiencing disability worsening (low to moderate), there is inconsistent evidence to recommend which therapies are most effective in preventing the worsening of disability. However, there was moderate confidence in the estimate of effect that Natalizumab statistically and clinically reduces the risk of disability worsening compared to a placebo at 24 months. Although most therapies were associated with a higher risk of treatment withdrawal because of an adverse event at 12 months, Fingolimod was the only treatment that had a significant risk at 24 months.

As the Cochrane review is now seven years old, it is important to consider more recent evidence in order to improve the understanding of the efficacy and safety of DMTs when treating RRMS. Further network meta-analyses have found Natalizumab and Alemtuzumab to consistently demonstrate a high ranking of efficacy across clinical outcomes including rate of relapse and disability progression (Fogarty et al, 2016; Lucetta et al, 2018; Li et al, 2020; Liu et al, 2021). Ocrelizumab has also been shown to have high efficacy among DMTs with a similar safety profile (Lucchetta et al, 2018; McCool et al, 2019; Li et al, 2020), and Ofatumumab was found to be superior to or as effective as other highly efficacious DMTs (Samjoo et al, 2020; Liu et al, 2021). Therefore, more recent findings support the results of the Cochrane review regarding the efficacy of Natalizumab and Alemtuzumab and go on to suggest that other DMTs, such as Ocrelizumab and Ofatumumab, may also be beneficial for reducing relapses and disability progression.

It should be noted that since the Cochrane review was published, Alemtuzumab has been reported by the European Medicines Agency to have rare but serious side effects, including cardiovascular and immune-related disorders, leading to restrictions on its use. Healthcare professionals are now advised that ‘*Lemtrada (alemtuzumab) should only be used to treat relapsing-remitting multiple sclerosis if the disease is highly active despite treatment with at least one disease-modifying therapy or if the disease is worsening rapidly. Lemtrada must also no longer be used in patients with certain heart, circulation or bleeding disorders or in patients who have autoimmune disorders other than multiple sclerosis* (European Medicines Agency, 2020). Therefore, recommendations for the use of specific DMTs should be based on an individualised approach to the benefits and harms for each patient, referring to National Institute for Health and Care Excellence (2022a) guidelines and National Institute for Health and Care Excellence (2022b) technology appraisal guidance for individual products.

When deciding on the use of DMTs, it is essential to consider all the relevant variables including efficacy, safety information (as described above), contraindications and how well patients will adhere to taking the therapy. In addition to individual contraindications, it has also been noted that live vaccinations may be contraindicated in people with MS who are being treated with DMTs and, thus, should be discussed with the patient (National Institute for Health and Care Excellence, 2022a). Medication adherence is also relevant to the decision-making process as adverse reactions, intolerance and disease activity ultimately result in treatment withdrawal (Yoon and Cheong, 2019). A study of medication adherence among patients with MS also identified that adherence to treatment was better with oral DMTs when compared with injectable DMTs (Lahdenperä et al. 2020).

A number of DMTs have been identified as beneficial for reducing the recurrence of relapses in RRMS. Most of the comparisons within this network meta-analysis however were based on a low number of trials and as such the results should be interpreted with caution. As the treatment of RRMS can be given for multiple decades, it is important that future trials of DMTs should ensure participants are followed up long-term. Given the limited evidence available for serious adverse events in this analysis, future trials should also ensure that a clear and transparent safety profile is captured and presented clearly.

## Figures and Tables

**Table 1:** Most effective therapies against the recurrence of relapses in relapsing-remitting multiple sclerosis during the first 12 months of treatment versus placebo

Ranking of effectiveness	Immunomodulator/immunosuppressant	Risk ratio 95% confidence interval (CI)	Surface under the cumulative ranking curve	Confidence in the evidence
1	Alemtuzumab	0.40, 95% CI: 0.31-0.51	97%	Moderate
2	Mitoxantrone	0.40, 95% CI: 0.20-0.76	93%	Low
3	Natalizumab	0.56, 95% CI: 0.43-0.73	85%	High
4	Fingolimod	0.63, 95% CI: 0.53-0.74	80%	Low

**Table 2:** Most effective therapies against the recurrence of relapses in relapsing-remitting multiple sclerosis during the first 24 months of treatment versus placebo

Ranking of effectiveness	Immunomodulator/immunosuppressant	Risk ratio 95% confidence interval (CI)	Surface under the cumulative ranking curve	Confidence in the evidence
1	Alemtuzumab	0.46, 95% CI: 0.38-0.55.	96%	Moderate
2	Mitoxantrone	0.47, 95% CI: 0.27-0.81	92%	Very low
3	Natalizumab	0.56, 95% CI: 0.47-0.66	88%	High
4	Fingolimod	0.72, 95% CI: 0.64-0.81	71%	Moderate

**Table 3:** Most effective therapies against worsening disability during the first 24 months of treatment

Ranking of effectiveness	Immunomodulator/immunosuppressant	Risk ratio 95% confidence interval (CI)	Surface under the cumulative ranking curve	Confidence in the evidence
1	Mitoxantrone	0.20, 95% CI: 0.05-0.84	96%	Low
2	Alemtuzumab	0.35, 95% CI: 0.26-0.48	94%	Low
3	Natalizumab	0.64, 95% CI: 0.49-0.85	74%	moderate
4	Azathioprine	0.64 95% CI: 0.30-1.37	64%	Very low

**Table 4:** Proportion of patients who withdrew from treatment (acceptability) because of an adverse event compared to placebo over 12 months

Ranking of risk (1=greatest risk)	Immunomodulator/immunosuppressant	Risk ratio 95% confidence interval (CI)
1	Fingolimod	8.26 95% CI: 3.25-20.97
2	Interferon beta-1a (Rebif)	4.83 95% CI: 2.59-9.00
3	Interferon beta-1a (Avonex)	4.36 95% CI: 1.98-9.60
4	Peg-interferon beta 1a	2.80 95% CI: 1.39-5.64
5	Teriflunomide	2.24 95% CI: 1.50-3.34

## Key points

- The most effective disease modifying therapies for preventing relapses in relapse-remitting multiple sclerosis, based on moderate to high evidence, are alemtuzumab, natalizumab and fingolimod.
- Reports of rare but serious side effects of alemtuzumab have led to restrictions on its use.
- There is inconsistent quality of evidence to state which therapies are most effective in preventing disability worsening.
- Several therapies had a significantly higher risk of treatment withdrawal because of an adverse event at 12 months.
- Safety information should always be considered alongside efficacy when determining the benefits and harm to an individual patient.
